# Mimicking women’s endocrine milieu in mice for women’s health-related studies

**DOI:** 10.1038/s44294-025-00060-4

**Published:** 2025-02-22

**Authors:** Céline Constantin, Daria Matvienko, Csaba László, Valentina Scabia, Laura Battista, Pierre-Alain Binz, Stephen J. Bruce, Cathrin Brisken

**Affiliations:** 1 Swiss Institute for Experimental Cancer Research, School of Life Sciences, Ecole Polytechnique Fédérale de Lausanne (EPFL), Lausanne, Switzerland; 2 Lonza AG, Visp, Switzerland; 3 PMI R&D Philip Morris Products S.A., Neuchâtel, Switzerland; 4 International Cancer Prevention Institute, Route de la Corniche 8, Epalinges, Switzerland; 5 Clinical Chemistry Laboratory, Centre Hospitalier Universitaire Vaudois (CHUV), Lausanne, Switzerland; 6 Institut Central des Hopitaux du Valais, Sion, Switzerland; 7 The Breast Cancer Now Toby Robins Breast Cancer Research Centre, The Institute of Cancer Research, London, UK

**Keywords:** Cancer, Physiology, Diseases, Cancer, Gynaecological cancer

## Abstract

To improve preclinical studies and their translation, patient-derived xenografts (PDXs) are increasingly used. They have human-specific tumor characteristics and reflect intra and inter-tumor heterogeneity. However, the endocrine milieu differs between humans and host mice. In light of sex-specific cancer biology and a rise in endocrine-related cancers there is an urgent need to correctly reflect the hormonal milieu in PDX models. We show that female mice of *NOD.Cg-Prkdc*^*scid*^ *Il2rg*^*tm1Wjl*^*/SzJ (NSG)* strain widely used for PDXs has 17-β-estradiol (E2) and testosterone (T) levels comparable to *C57Bl6* females but higher progesterone (P4) levels. E2 levels are comparable, T levels are lower and P4 levels higher than those observed in postmenopausal women. Ovariectomy increases T to levels observed in postmenopausal women. Subcutaneous E2 and combined E2/P4 silicon pellets provide *NSG* females with premenopausal ovarian hormone levels. These procedures humanize the endocrine environment of experimental animals, improving PDX relevance in women’s health-related research.

## Introduction

Research into women’s health depends on experimental models ranging from in vitro cell lines to complex genetically engineered mouse and xenograft models^1–4^. Notably, in oncology, the success rate of clinical translation has been low because the models used in preclinical research fail to adequately mimic human disease^5–7^. To increase translatability, PDXs are increasingly used because they capture intra- and inter-tumor heterogeneity better than the widely used cell lines or genetically engineered mouse models^2,4,8,9^. Improved engraftment by injection into the milk ducts has recently been shown to be enabling for studying both normal breast epithelial cells as well as breast cancer cells^10–12^ when in particular, estrogen receptor-positive breast cancer was previously difficult to study^13–15^.

Host mice are severely immunocompromised^16^ allowing them to tolerate grafts of human origin but also presenting a major limitation of xenograft models. In recent years, numerous efforts to humanize the immune system of recipient mice have further improved the predictive value of xenograft studies^17–22^. Yet, another important factor, the hormonal milieu of the models used, is often overlooked.

Ovarian hormones control numerous physiological functions and impinge on tumor development^23–25^. While this is well established for breast cancer and tumors of other reproductive organs, evidence has accumulated that this applies also to malignancies arising from non-reproductive organs^26,27^. For instance, bladder cancer has a higher incidence in men than in women and the role of androgen receptor signaling in its progression is actively explored^28–30^. Similarly, higher incidence rates of hepatocellular carcinoma in males depend on sex hormones, with estrogen receptor signaling suppressing and androgen receptor signaling promoting hepatocarcinogenesis^31,32^. Likewise, E2 plays a role in thyroid cancer, which primarily affects women^33–35^. Furthermore, epidemiologic studies have revealed that early menarche along with late menopause and hormone replacement therapy increase the risk of various hormone-dependent tumors^36–40^.

While most hormones are shared across mammals, there are differences among species with regard to reproductive biology, hormone levels, and their fluctuations. Female mice have approximately 4- day reproductive cycles known as estrous cycles consisting of 4 stages, proestrus, estrus, metestrous, and diestrus while the human menstrual cycles last between 21 to 35 days and are divided into follicular and luteal phase^41^. Humans are one of few mammalian species who experience menopause, a reproductive stage that is not observed in rodents. Additionally, there are differences in mouse and human sex steroid metabolism. In humans, the plasma protein, sex hormone binding globulin (SHBG), binds with high affinity to sex steroids and controls their availability by regulating their tissue distribution and metabolism^42^. Rodents do not express this protein and this affects how the sex hormones circulate and function. Similarly, in humans, but not rodents, the adrenal gland secretes substantial amounts of the sex steroid precursors, androstenedione and dehydroepiandrosterone^43^. Together, these differences result in species-specific plasma concentrations of sex steroids^44^.

To gain physiologically relevant insights into female health, sex steroid profiles need to be adequately reflected in the experimental design^45–47^. The challenges in obtaining reliable data about endocrine profiles largely stem from the limitations of the traditional steroid hormone immunoassays, which often suffer from low sensitivity and lack of specificity. This is due to the cross-reactivity of structurally similar steroid precursors and metabolites^48,49^. Additionally, it has been difficult to measure hormone levels in mice due to the high sample volume requirement. The low steroid hormone concentrations and the inability to measure more than one hormone at a time with immunoassays were additional challenges^48–51^. Mass spectrometry (MS) is ideally suited for analyzing sex steroids in plasma samples because multiple substances can be measured simultaneously with high precision and accuracy^44,52,53^. Currently, liquid chromatography (LC)-MS is widely regarded as the gold standard for analyzing steroid hormones. However, successful implementation of LC-MS in hormone analysis requires careful setup and continuous maintenance of equipment, as well as expert interpretation of the raw data. This, along with their operational running costs represents significant investments. Additionally, the analysis requires running numerous samples concomitantly to ensure data are robust and appropriate controls to validate results are incorporated. Moreover, previous studies using LC-MS and gas chromatography–mass spectrometry (GC-MS) have faced challenges in detecting E2 in murine models^50,54^ with levels in the lower picomolar range.

With the present study, we address the need for more physiologic models in the study of hormone-sensitive diseases. We present an LC-MS assay to measure concomitantly E2, estrone (E1), P4, and T in a single small volume plasma sample as can be routinely obtained by tail vein bleeding of small rodents and measure these in female *NSG* mice, the most widely used immunocompromised mouse strain in PDX modeling. Finally, we propose simple approaches to mimic critical physiological stages in women’s lives to enable more physiologically relevant modeling of women’s health issues.

## Results

### LC-MS method to measure E2, E1, P4, and T

**Fig. 1:**
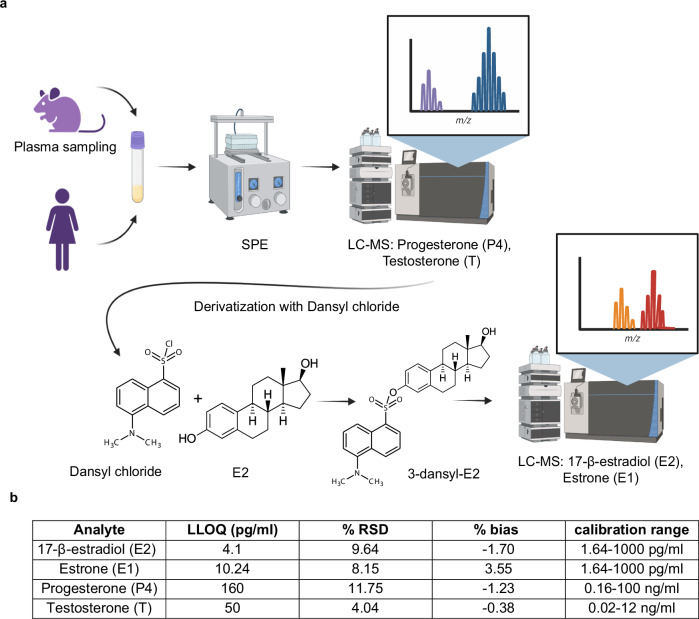
LC-MS-based analysis of major ovarian hormone plasma levels. **a** Illustration of the workflow of the optimized LC-MS method for simultaneous measurements of four ovarian hormones in plasma samples from humans and mice as small as 100 μl. Plasma samples are prepared using solid-phase extraction (SPE) for LC-MS analysis of P4 and T. The remaining samples are derivatized and analyzed by LC-MS for E2 and E1. Created in BioRender. Matvienko, D. (2024) BioRender.com/h27d558 with modifications. **b** Table showing lower limits of detection (LLOQ), % Relative standard deviation (RSD), % bias at LLOQ and calibration ranges for the different analytes, E2, E1, P4, and T.

To measure the ovarian hormones in a single blood sample from small animals, we adapted an LC-MS method for progestins and steroids^53,55^. To enable the detection of estrogens, we introduced a derivatization step with dansyl chloride to improve the ionization efficiency through reaction with their phenolic hydroxyl group^56–58^ (Fig. 1a). Sensitivity evaluation revealed that the lower limits of quantitation (LLOQ) for E2, E1, P4, and T from 100 μl of plasma were 4.1, 10.24, 160, and 50 pg/ml, respectively (Fig. 1b). Thus, 100 µl of plasma as obtained through routine tail bleeding are sufficient to measure four major ovarian hormones facilitating routine profiling in small animal experiments.

### Plasma levels of ovarian steroids in *NSG* mice

**Fig. 2:**
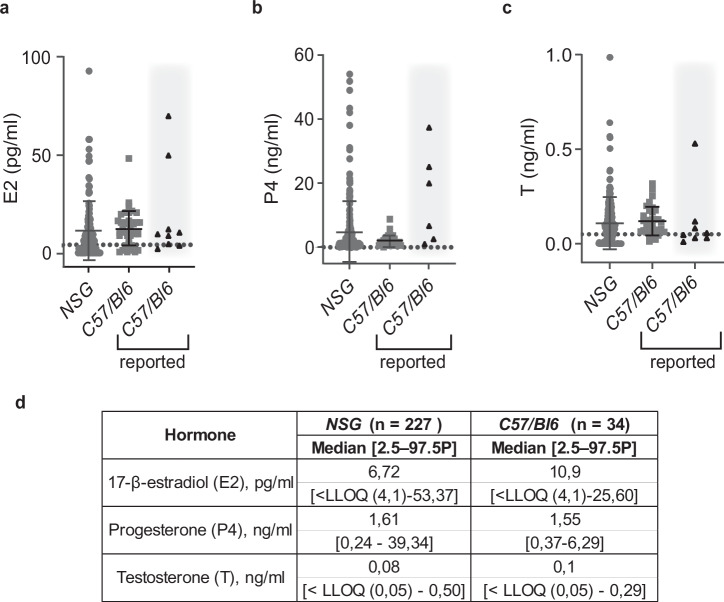
Ovarian hormone plasma levels in *NSG* and *C57/*Bl6 adult females. **a** Dot plot showing E2 plasma levels in *NSG* females. **b** Dot plot showing P4 plasma levels in *NSG* females. **c** Dot plot showing T in *NSG* females. The plasma levels in 10 to 20-week-old *NSG* females (gray circles) are compared to levels measured by us in *C57/Bl6* mice (gray squares)^55^. Each dot represents an individual mouse, and bars represent the median hormone levels with error bars indicating the range (means ± SD). Black triangles on gray background indicate upper and lower ranges for *C57/Bl6* strain previously reported by others^44,59–65^. Grid lines indicate LLOQ. *NSG* *n* = 227, *C57/Bl6* *n* = 34. No significant differences were observed between the groups (Mann-Whitney *U* test, *p* > 0.05). **d** Table showing median plasma concentrations and 2.5–97.5th percentiles for 10 to 20-week-old females of *NSG* and *C57/Bl6* background. Note that E1 levels were below LLOQ.

*NSG* mice are widely used in biomedical research for xenograft experiments because of their broad immune suppression. Yet, there are no reference values of their hormone plasma levels. We determined E2, E1, P4, and T plasma concentrations in adult virgin *NSG* females and compared them to those of *C57/Bl6* mice we reported previously^55^ as well as to values from other MS-based studies^44,59–65^. E2 plasma levels in *NSG* females with mean values of 11.21 pg/ml were comparable to *C57/Bl6* females with a mean value 12.41 pg/ml (Fig. 2a, d). P4 levels tended to be higher and more variable in *NSG* mice with a mean of 4.90 ± 9,26 ng/ml compared to P4 levels of *C57/Bl6* mice in our dataset with mean of 2.11 ± 1.73 ng/ml. The levels were within the range of published values for *C57/Bl6* mice^44,59–63^ (Fig. 2b, d). T levels were similar in both strains with mean 0.10 ng/ml in *NSG* and 0.12 ng/ml in *C57/Bl6* mice (Fig. 2c, d). Most E1 measurements were below the LLOQ in both strains (data not shown). Thus, *NSG* and *C57/Bl6* females have comparable sex steroid profiles. While our analysis focused on ovarian hormones, additional measurements of corticoid steroids—corticosterone (B) and deoxycorticosterone (DOC) were performed on the same mouse strains and no significant differences were observed between the strains (Supplementary Fig. 1).

### Plasma ovarian steroid levels in murine versus human samples

**Fig. 3:**
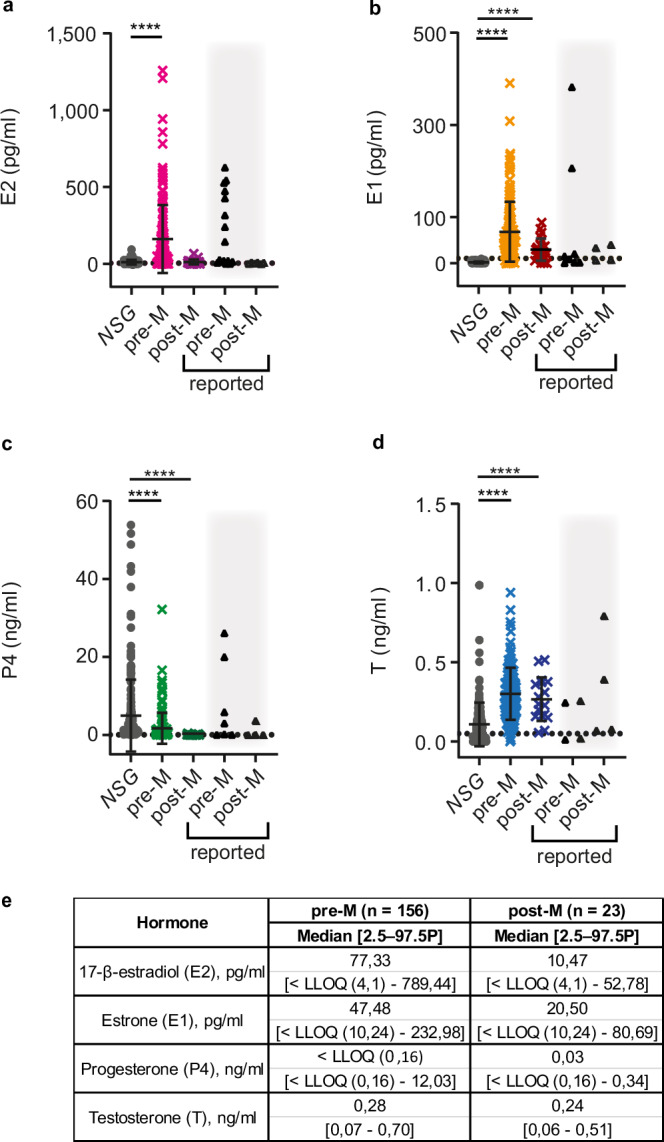
Endogenous ovarian steroid plasma levels of *NSG* females and pre- and postmenopausal women. **a** Dot plot showing E2 plasma levels in *NSG* females (gray circles), premenopausal women (pre-M) (magenta crosses) and postmenopausal women (post-M) (violet crosses). **b** Dot plot showing E1 plasma levels in *NSG* females (gray circles), pre-M women (bright orange crosses) and post-M women (red crosses). **c** Dot plot showing P4 plasma levels in *NSG* females (gray circles), pre-M women (green crosses) and post-M women (dark green crosses). **d** Dot plot showing T plasma levels in *NSG* females (gray circles), pre-M women (blue crosses), and post-M women (dark blue crosses). Each dot represents an individual sample and bars represent means ± SD. Black triangles on gray background indicate previously reported upper and lower ranges for pre- and postmenopausal women^66–70^. Grid lines indicate LLOQ. *NSG* *n* = 227, pre-M *n* = 156, post-M *n* = 23. Statistical significance was tested using Kruskal-Wallis One-Way ANOVA comparison test, **** indicates *p* < 0.0001. **e** Table showing median plasma concentrations and 2.5–97.5th percentiles for pre- and postmenopausal women.

To compare the ovarian hormone levels detected in *NSG* females to those observed in women, we measured the 4 ovarian steroids in plasma from 156 pre- and 23 post-menopausal women by the same approach and recurred to published reference values^66–70^. Murine E2 levels with mean of 11.21 pg/ml were more than 10-fold lower than those observed in premenopausal women who had mean values of 161 pg/ml and comparable to those in postmenopausal women with mean of 12.80 pg/ml (Fig. 3a, e). E1 was readily detected in the plasma of both pre- and post-menopausal women with mean values of 67.85 and 29.32 pg/ml respectively, while below LLOQ with our method in mice (Fig. 3b, e). Plasma P4 levels (mean 4.90 ng/ml) were higher in mice than in pre- and post-menopausal women with mean values 1.70 and 0.07 ng/ml respectively (Fig. 3c, e). The mean P4 value observed in *NSG* mice was comparable to luteal phase levels in women, which range from ~5 to 20 ng/ml (Fig. 3c, e). T levels with mean 0.10 ng/ml were lower in mice than in pre-and postmenopausal women with means of 0.30 and 0.26 ng/ml, respectively (Fig. 3d, e). The E2 and P4 concentrations measured in women from our cohort were in the same range as the reported reference values^66–70^ (Fig. 3a, c). However, the range of E1 levels with a mean 29.32 pg/ml in postmenopausal women we measured (Fig. 3b, e) was twice as wide as ranges reported by others^44,71–73^ (Fig. 3b). Taken together, murine E2 levels are comparable to those found in postmenopausal women while murine P4 levels are closer to luteal phase values in humans.

### Hormone treatments of mice mimic pre- and postmenopausal steroid profiles

To mimic the endocrine milieu of pre- and postmenopausal women, respectively, we used different strategies—subcutaneous implantation of slow-release hormone pellets with E2, P4, or E2 and P4 combined or hormone ablation by ovariectomy^12^.

**Fig. 4:**
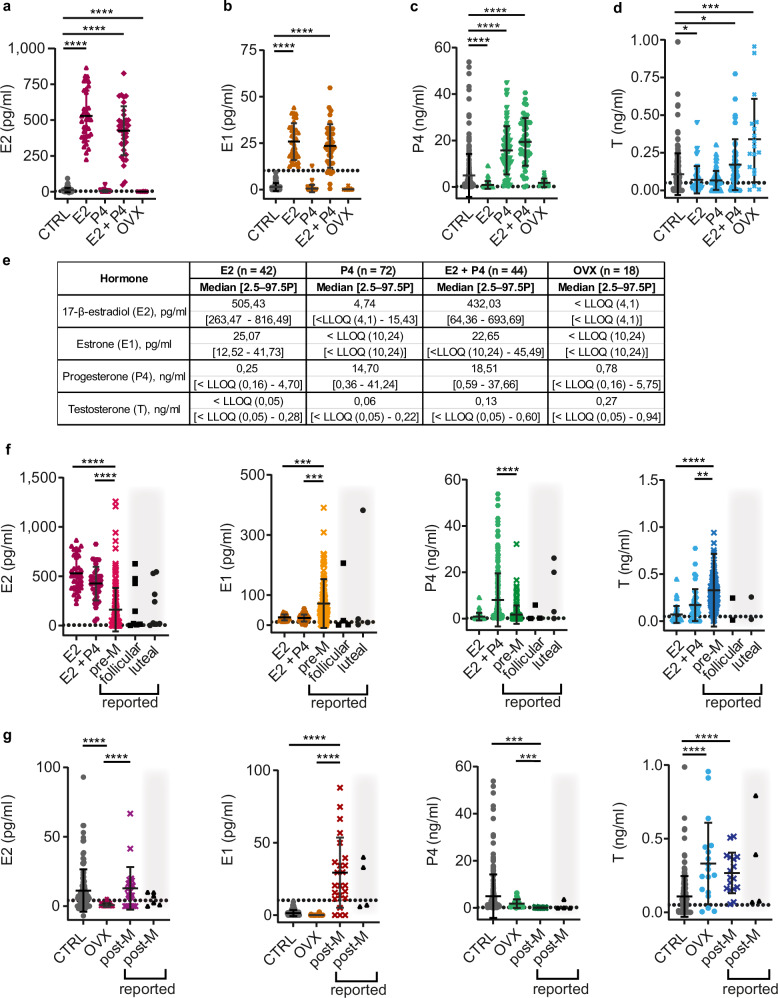
Ovarian steroid levels in *NSG* females following hormone treatments. **a** Dot plot showing E2 plasma levels in *NSG* females: control (CTRL) (gray circles), E2-, P4-, E2 + P4-treated, and ovariectomized (OVX) (dark magenta circles) mice after 60-day treatment. **b** Dot plot showing E1 plasma levels in *NSG* females: CTRL (gray circles), E2-, P4-, E2 + P4-treated, or OVX (orange circles) after 60-day treatment. **c** Dot plot showing P4 plasma levels in *NSG* females: CTRL (gray circles), E2-, P4-, E2 + P4-treated, or OVX (light green circles) after 60-day treatment. **d** Dot plot showing T plasma levels in *NSG* females: CTRL (gray circles), E2-, P4-, E2 + P4-treated, or OVX (light blue circles) after 60-day treatment. **e** Table showing median plasma concentrations and 2.5–97.5th percentiles for treated mice. **f** Dot plots comparing plasma levels of E2, E1, P4, and T in *NSG* females treated for 60 days with E2- and E2 + P4 pellets to premenopausal hormonal levels in women (pre-M); color codes correspond to (**a**–**d**) and Fig. 3a–d. **g** Dot plots comparing plasma levels of E2, E1, P4, and T in CTRL and OVX *NSG* females to postmenopausal hormonal levels in women (post-M); color codes correspond to (**a**–**d**) and Fig. 3a–d. Each dot represents an individual mouse and the bars represent the mean hormone levels with error bars indicating ± SD. Black triangles on gray background indicate previously reported upper and lower ranges by others for follicular and luteal phase or post-menopausal women^66,68,70^. Grid lines indicate the LLOQ. CTRL *n* = 227, E2 n = 42, P4 *n* = 72, E2 + P4 *n* = 44, OVX *n* = 18, pre-M *n* = 156, post-M *n* = 23. Statistical significance was determined using Kruskal–Wallis One-Way ANOVA comparison test, * indicates *p* < 0.05, ** *p* < 0.01, *** *p* < 0.001, **** *p* < 0.0001.

Sixty days after the implantation of 0.3 mg E2-containing silicone pellets, E2 plasma levels increased 46-fold with mean 529.30 pg/ml compared to intact mice (mean 11.21 pg/ml) (Fig. 4a, e). Consistent with a fraction of the administered E2 being converted to E1, E1 levels increased 17-fold with mean 25.82 pg/ml (Fig. 4b, e). P4 levels were decreased to mean 0.85 pg/ml (Fig. 4c, e) consistent with exogenous E2 suppressing the estrous cycle-related peaks in P4 levels^74^. The 20 mg slow-release P4 pellets increased plasma P4 levels 2-fold with mean 15.75 ng/ml compared to intact mice (mean 4.90 ng/ml) and had no effect on other hormone levels (Fig. 4c, e).

In premenopausal women, E2 levels peak during the follicular phase, while the luteal phase is characterized by a lower peak in E2 levels and high P4 levels. To mimic human luteal phase hormone levels and overcome the inhibitory effect of E2 treatments on ovarian P4 secretion, we implanted mice with 0.3 mg E2 pellets alongside 20 mg P4 pellets and compared the resulting hormone levels to published data^66,68,70^. E2 levels were higher in treated mice with mean 426.30 pg/ml than in premenopausal women (mean 161 pg/ml) and had higher P4 with mean 19.39 ng/ml than premenopausal women (mean 1.70 ng/ml) (Fig. 4f). It is important to note that we did not separate the plasma values from women of our dataset by phase of the menstrual cycle, which results in more heterogeneous hormone ranges. E1 and T remained lower in mice than in women (Fig. 4f). Thus, E2- and combined E2 + P4-treated mice have E2 and P4 plasma levels comparable to those in women during follicular and luteal phase^66,68,70^, respectively.

Intact mice have similar E2 (mean 11.21 pg/ml), but higher P4 levels (mean 4,90 ng/ml) than postmenopausal women (means E2: 12.80 pg/ml and P4: 0.07 ng/ml) (Fig. 3a, c and Fig. 4g). Therefore, we compared the steroid profile of ovariectomized mice with decreased E2 and E1 levels to postmenopausal women. Sixty days following ovariectomy, both E2 and E1 levels dropped below the quantitation limit (Fig. 4a, b, g). Plasma P4 levels in ovariectomized mice remained higher with a mean value 1.74 ng/ml compared to postmenopausal patients, though these levels were closer to human settings than those observed in intact mice (Fig. 4g). Ovariectomy elevated mouse plasma T concentrations to mean 0.33 ng/ml, thereby better matching levels of postmenopausal women (mean 0.27 ng/ml) (Fig. 4g). Thus, postmenopausal E2 levels are similar to levels in intact *NSG* females, but T and P4 levels are better mimicked in ovariectomized mice.

## Discussion

Mouse models are essential in preclinical and translational research, and improving their predictive power by better mimicking human physiology is a continuous effort. Here, we sought to improve the endocrine context of the murine models, with regard to ovarian steroid hormones, central to women’s health. We focused on the widely used *NSG* strain and proposed a simple practical approach to mimic distinct endocrine milieus pertinent to women’s health. First, we optimized an LC-MS method^53,55^ for the simultaneous measurement of E2, E1, P4, and T in 100 µl of plasma. This optimization is crucial for working with low-volume mice blood samples and allows for repeat measurements via tail vein sampling vital for accurately monitoring hormone fluctuations over time. Our LC-MS method, while effective, has limitations; like higher LLOQs than other methods such as GC-MS^44^. This applies in particular to E1, where the values we measured remained below the quantitation limit in untreated mice and higher errors for low values may also explain the high variation we observed in plasma samples from postmenopausal women. Additionally, tandem LC–MS/MS with triple quadrupole type instruments provide a wide range from LLOQs with a minimum of 0.14 pg/ml for E1 and E2^75^ providing potential for further improvement of the present method.

Our findings demonstrate that intact *NSG* mice have plasma E2 concentrations similar to postmenopausal women and suggest that at least with regard to E2 levels they provide an approximative endocrine milieu of postmenopausal women, whereas T and P4 levels were better reflected by ovariectomized mice. Interestingly, while T levels increase upon ovariectomy in *NSG* mice, they decrease in oophorectomized women^76^. This may be an *NSG*-specific phenomenon as reduced but still measurable T levels were observed in ovariectomized *C57/BL6* mice and suggest that the adrenal glands may contribute to circulating T levels in female mice^44^. Further studies are needed to determine if the adrenal glands can compensate for T production in the case of gonadectomy. Moreover, the day of the estrous cycle when the blood sample is collected may influence T hormone levels^77^. As *NSG* mice lack B and T cells, the estrous cycle stage could not be assessed by vaginal smear. The present method being simple to implement, will enable routine measurements in experimental settings allowing researchers to acquire more information about hormone levels and how different treatments may affect them in the future. Our measurements of corticoid steroids, albeit beyond the focus of the current analysis, provide some further information about the hormonal environment. Specifically, with the use of the same LC-MS approach, we found the two major rodent corticoid hormones B and DOC to be comparable in *NSG* and *C57BL/6* suggesting that they may not play a critical role in strain-specific differences. How the biological impact of these levels relates to the activities of cortisol, the major corticosteroid found in women, remains to be addressed.

To better match the hormone profile of women with murine models, we propose simple strategies. The approaches are low cost, the fabrication of slow-release hormone pellets is flexible and readily adjustable to different time windows requirements, moreover it can readily be extended to other steroids.

The E1 levels were consistently lower in mice than humans, regardless of treatment. None of the strategies we tested allowed to match E1 levels in postmenopausal women without simultaneously increasing E2. To mimic the specific sex steroid levels in a murine model, this issue could be addressed by using E1 pellets can be added^78^. Additionally, since estrogens can inhibit the hypothalamic secretion of gonadotropin-releasing hormone^79,80^ E1 pellets may also help suppress the cycle-related variation in circulating P4. As such, ovariectomized mice with E1 pellets might most accurately reflect the postmenopausal endocrine milieu.

E2 and combined E2 and P4 slow-released pellets allowed us to mimic the follicular and luteal phase levels of the two major ovarian hormones. Our results showed that E2 levels in treated mice were higher compared to premenopausal women which is likely due to the use of a single concentration of E2 pellet of 0.3 mg in our study. Adjusting the pellet dosage by titration could help to better approximate the E2 levels observed in premenopausal women, improving the translational relevance of our model. An important limitation of hormone-released pellet implantation is that hormone release decreases over time and fails to mimic natural cyclicity. Slow-release silicone-based implants are easy to prepare and are flexible, but they administer chronic instead of fluctuating doses of hormones. Drug delivery technology devices for more accurate and continuous delivery are commercially available and more drug-release devices with varying release rates are currently under development^81–84^. Depending on the specific research question, scientists may choose to use hormone treatments to more accurately mimic patients’ physiology. With the present approach, flexible tools are at hand.

## Methods

### Animal experiments

All animal experiments were performed in accordance with the Animal Experimentation Ordinance (SR 455.163) and authorized by the Direction des Affaires vétérinaires et de l’inspectorat (DAVI) in Canton Vaud (VD 1865.3, 1865.4, and 1865.5). All procedures adhered to the ARRIVE guidelines for animal experimentation. *NOD.Cg-Prkdc*^*scid*^ *Il2rg*^*tm1Wjl*^*/SzJ* mice (*NSG*) were purchased from Jackson Laboratories. All animals were maintained in the EPFL animal facility (VD-H11) in groups of up to five mice in individually ventilated cages (GM500, Tecniplast) with a 12 h light-12 h dark cycle at 20–24 °C with 45–65% humidity in accordance with the Animal Welfare Act (SR 455), and the Animal Welfare Ordinance (SR 455.1). The water was acidified to a pH of 2.5–3 using a resin column (Prominent® CH system), and the diet consisted of irradiated feed from Provimi-Kliba® (cat# 3242). 10–20-week-old female mice, previously engrafted intraductlly with either normal human breast epithelial cells or ER+ breast cancer cells^10,11^ were randomly assigned to experimental groups and implanted with slow-release hormone pellets. Only healthy animals were selected for the experiment to ensure reliable and consistent results. The exact number of experimental animals allocated to each experimental group is reported in the figure legends. Experimenters were blinded to the group assignments during data collection and analysis to prevent bias. During the implantation of hormone pellets, mice were anesthetized using 2–2.5% isoflurane with oxygen, administered via an inhalation mask. Isoflurane was selected for its rapid onset and minimal recovery time. The depth of anesthesia was monitored by assessing reflexes and respiratory rate. Paracetamol (200–300 mg/kg) was administered in drinking water starting one day before surgery and continued for 3–5 days post-surgery to provide ongoing analgesia. For local analgesia, a mix of Lidocaine (10 mg/kg) and Bupivacaine (2.5 mg/kg) was injected subcutaneously three minutes before the incision at the site of the future incision to ensure effective numbing of the area. During the surgery, the mice were placed on a heating pad to maintain body temperature and minimize post-anesthetic hypothermia until they were fully awake to ensure a smooth recovery. Sixty days post pellet implantation, blood was sampled through tail vein or heart puncture. For tail vein blood collection, no anesthesia was applied, and mice were maintained conscious. For heart puncture, animals were euthanized via intraperitoneal injection of xylazine (10 mg/kg) and ketamine (75 mg/kg). Reflexes and respiration were monitored to ensure animals were unconscious before the procedure. The xylazine/ketamine cocktail was chosen for its effectiveness in providing deep anesthesia and analgesia. In adherence to the principles of the 3Rs (Replacement, Reduction, and Refinement), animals used in this study were reutilized from other experiments conducted in the lab and were not specifically euthanized for the purpose of blood collection. A detailed ARRIVE guideline checklist, ensuring transparent reporting of the study’s conduct, and analysis, is provided in Supplementary File 1.

### Patient samples

Human plasma samples were obtained from patients undergoing breast reduction surgery at the Centre Hôspitalier Universitaire Vaudois and Hirslanden Hospital. The study was approved with all relevant ethical regulations including the Declaration of Helsinki by the Commission cantonale d’éthique de la recherche sur l’être humain ethics committee (VD183/10) and informed consent was obtained from all the participants. Patients on hormonal contraception or hormone replacement therapy and blood samples, in which a progestin was detectable by LC-MS (gestodene, levonorgestrel, etonogestrel, chlormadinone acetate, cyproteroneacetate, drospirenone, desacetyl norgestimate, medroxyprogesterone acetate, norethindrone, dienogest or nomegestrol acetate) were excluded^53^.

### Pellet preparation

Pellets were prepared by mixing parts A (MP3745/E81949) and B (MP3744/E8195) of the low-viscosity silicon elastomer (MED-4011) with hormone powder as indicated below (Table 1). The mix was incubated overnight at 37 °C and cut as described^12^.

**Table 1:** Hormonal pellet composition and dosage details

Hormone	Catalog number	Hormone dose/pellet (mg)	Silicon part A (mg)	Silicon part B (µl)	Hormone powder (mg)	Pellet weight (mg)
17β-estradiol (E2)	E2758	0.3	4700	500	250	7.8
Progesterone (P4)	P0130-25G	20	3525	375	3525	49.5

Amounts of individual components used for individual silicon pellets of E2 and P4 were used in the study. Indicated are hormone dose per pellet in milligrams (mg), silicon part A (mg), and volume of part B in microliters (µl), amount of hormone powder (mg), and pellet weight (mg).

### Hormone measurements

E2, E1, P4, and T as well as B and DOC plasma levels were measured by high-resolution LC-MS (Q-Exactive Orbitrap, ThermoFisher Scientific) using targeted-SIM (tSIM) acquisition^53,55^ with the following modifications. For sample preparation, 100 µl of plasma were mixed with 100 µl of internal standards in 5% (w/v) phosphoric acid, applied to a solid phase extraction plate (Oasis MCX µElution 96-well plate, Waters) and washed with 5% (w/v) ammonium hydroxide (NH_4_OH) and 20% (v/v) methanol and eluted with isopropyl alcohol. Eluates were evaporated under N_2_ on a TurboVap 96 (Biotage, Uppsala, Sweden) and reconstituted in 100 µl 40% acetonitrile (ACN), corresponding to initial LC mobile phase conditions. To detect P4 and T, 20 µl of the sample were injected onto a Zorbax Eclipse Plus C18 (2.1 × 50 mm 1.8 µ m) column (AgilentTechnologies, Santa Clara, California, United States) at a flow rate of 0.2 ml/min using a gradient of purified H_2_O and ACN, both containing 0.1% (v/v) formic acid. Solvent gradient was started at 40% ACN, held for 2 min, linearly increased to 70% ACN over 6 min, further increased to 100% ACN over 0,5 min, held at 100% ACN for 1 min, decreased to 40% ACN over 0.5 min and finally held at 40% ACN for re-equilibration until end of 12 min run. For the combined analysis of E2 and E1 with P4 and T, the same sample extracts were again evaporated under nitrogen and reconstituted in 50 µL sodium bicarbonate buffer (NaHCO_3_, 100 mM, pH adjusted to 10 with NaOH) and 50 µl dansyl chloride (2.0 mg/ml in acetone) were added per sample for derivatization. The plate was left at 65 °C for 15 min in an incubator, cooled to 4 °C and transferred to the LC autosampler. For E2 and E1 detection, 25 µl of sample were injected into the same LC column at a flow rate of 0.2 ml/min using a gradient of purified H_2_O and ACN, both containing 0,1% (v/v) formic acid. The solvent gradient started at 70% ACN and was held for 2 min, linearly increased to 85% ACN over 6 min, further increased to 100% ACN over 2 min, held at 100% ACN for 1 min, decreased to 70% ACN over 1 min and finally held at 70% ACN for re-equilibration until the end of the 15 min run. The mass spectrometer was a QExactive Orbitrap working in positive mode using previously reported parameters (Laszlo et al. ^53^) for P4 and T and the following modifications for E2 and E1: resolution 70k, automatic gain control (AGC) target 1e6, MAX ion injection time 100 ms, isolation window 0.5 m/z, scan range 500–750 m/z. The tSIM method acquires the user-defined targeted masses in retention time-dependent time segments. Data were processed with Thermo Xcalibur 4.0.27.10. The targeted SIM method contained an inclusion list with 10 ppm precision (Supplementary Table 1). Mass extraction for analysis was carried out at 5 ppm. LLOQs were determined on triplicate samples serially diluted 1:2.5 over eight dilutions.

### Statistical analysis

Statistical analysis was performed using GraphPad Prism (version 10) (San Diego, California, USA, www.graphpad.com). For datasets that included values below the limit of quantification (LLOQ), the Substitution Method was applied, in which values below the LLOQ were substituted with half the LLOQ (LLOQ/2). Normality was assessed using the Shapiro-Wilk test. For comparisons between two independent groups, the Mann-Whitney *U* test was used. For comparisons involving more than two groups, the Kruskal-Wallis test was employed. Statistical tests are indicated in the figure legends. All statistical tests were two-tailed, and statistical significance was defined as *p* < 0.05. Data are presented as mean ± standard deviation (SD).

## Supplementary information

Supplementary Information

## Data Availability

Data is provided within the manuscript. The raw data that support the findings of this study are available from the corresponding author upon reasonable request.
